# Surgical outcomes of pulmonary metastasectomy in hepatocellular carcinoma patients according to approach method: thoracoscopic versus open approach

**DOI:** 10.1186/s12957-021-02138-0

**Published:** 2021-01-30

**Authors:** Han Pil Lee, Jae Kwang Yun, Hee Suk Jung, Duk Hwan Moon, Geun Dong Lee, Sehoon Choi, Yong-Hee Kim, Dong Kwan Kim, Seung Il Park, Hyeong Ryul Kim

**Affiliations:** 1 Department of Thoracic & Cardiovascular Surgery, Kangwon National University Hospital, Kangwon National University College of Medicine, Chuncheon, Republic of Korea; 2 Department of Thoracic & Cardiovascular Surgery, Asan Medical Center, University of Ulsan College of Medicine, Seoul, Republic of Korea; 3 Department of Thoracic and Cardiovascular Surgery, CHA Bundang Medical Center, CHA University, Seoul, Republic of Korea; 4 Department of Thoracic and Cardiovascular Surgery, Gangnam Severance Hospital, Yonsei University College of Medicine, Seoul, Republic of Korea

**Keywords:** Hepatocellular carcinoma, PM, Metastasectomy, Video-assisted thoracoscopic surgery (VATS)

## Abstract

**Background:**

The role of surgical intervention as a treatment for pulmonary metastasis (PM) from hepatocellular carcinoma (HCC) has not been established. In this study, we investigated the clinical outcomes of pulmonary metastasectomy. Using propensity score matching (PSM) analysis, we compared the results according to the surgical approach: video-assisted thoracic surgery (VATS) versus the open method.

**Methods:**

A total of 134 patients (115 men) underwent pulmonary metastasectomy for isolated PM of HCC between January 1998 and December 2010 at Seoul Asan Medical Center. Of these, 84 underwent VATS (VATS group) and 50 underwent thoracotomy or sternotomy (open group). PSM analysis between the groups was used to match them based on the baseline characteristics of the patients.

**Results:**

During the median follow-up period of 33.4 months (range, 1.8–112.0), 113 patients (84.3%) experienced recurrence, and 100 patients (74.6%) died of disease progression. There were no overall survival rate, disease-free survival rate, and pulmonary-specific disease-free survival rate differences between the VATS and the open groups (*p* = 0.521, 0.702, and 0.668, respectively). Multivariate analysis revealed local recurrence of HCC, history of liver cirrhosis, and preoperative alpha-fetoprotein level as independent prognostic factors for overall survival (hazard ratio, 1.729/2.495/2.632, 95% confidence interval 1.142–2.619/1.571–3.963/1.554–4.456; *p* = 0.010/< 0.001/< 0.001, respectively).

**Conclusions:**

Metastasectomy can be considered a potential alternative for selected patients. VATS metastasectomy had outcomes comparable to those of open metastasectomy.

## Introduction

Hepatocellular carcinoma (HCC) is one of the most frequent malignancies in Asia, including Korea [1], and ranks third in cancer deaths worldwide [2]. In the recent years, the postoperative morbidity and mortality rates from HCC have improved; however, long-term outcomes remain poor due to extrahepatic metastasis and intrahepatic recurrence after surgical management. The most frequent site of metastasis and of the first detectable metastasis was reported to be the lungs [3, 4]. However, appropriate management for pulmonary metastasis (PM) has not been established, and systemic chemotherapy has been reported to be largely ineffective [5]. The results of the multinational, randomized, placebo-controlled, phase III sorafenib HCC Assessment Randomized Protocol (SHARP) trial demonstrated that sorafenib significantly improved the overall survival in patients with advanced HCC and well-preserved liver function, and that drug-related adverse events were manageable [6]. However, according to the subanalysis of phase II sorafenib Asia-Pacific trial, the effectiveness of sorafenib in advanced HCC patients remains unclear as the survival gain in patients with PM was only 1.4 months (hazard ratio [HR] 0.87) [7]. Although sorafenib has been a standard regimen for advanced HCC, its effectiveness is marginal in the presence of PM. In most cases, the lesions of extrahepatic metastasis from HCC are not resectable. Therefore, surgical treatment of PM has not been applied as a standard therapy in PM from HCC.

In some reports, pulmonary resection for PM from HCC resulted in long-term survival in highly selected patients [8–10]. Usually, wedge resection through open thoracotomy has been performed for superficial lesions, and segmentectomy or lobectomy is needed for deeper lesions. With recent advances in VATS and diagnostic modalities, such as multi-detector row computed tomography (CT), minimally invasive and complete metastasectomy can be provided to chronically ill patients with HCC and PM [1–14]. However, the use of surgical resection and VATS for PM of HCC remains controversial. We believe that an appropriate surgical role is needed for the treatment of patients with advanced HCC. In this study, we investigated the clinical outcomes of pulmonary metastasectomy and the risk factors for survival rate and disease-free survival rate in patients of HCC with PM. Using propensity score matching (PSM) analysis, we compared the results according to surgical approach: VATS versus open thoracotomy or sternotomy methods.

## Methods

### Patients

**Fig. 1:**
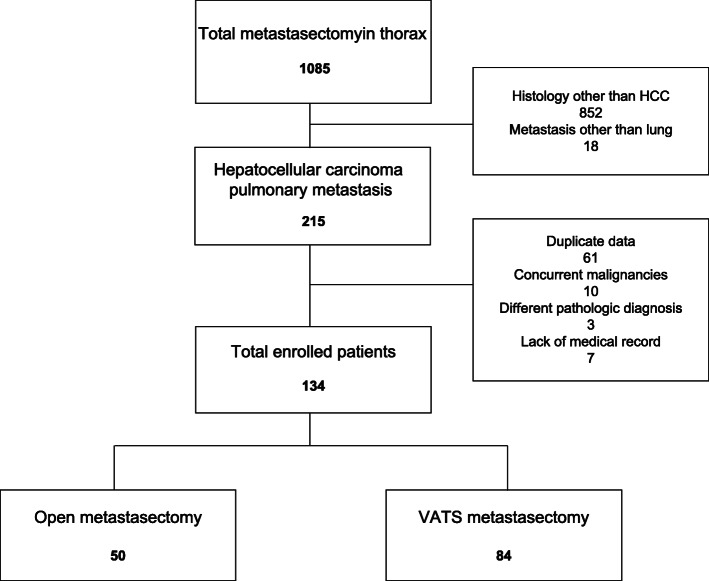
The enrollment of patients

In this study, we retrospectively reviewed a total of 1085 patients who had undergone metastasectomy due to thoracic metastasis from HCC between January 1998 and December 2010. Of these, 852 cases with different histology and 18 cases with metastatic lesions in the thorax other than the lung were excluded. Sixty-one cases were further excluded due to duplicated patients. Ten patients had concurrent malignancies, and 3 cases were diagnosed with a different histology from HCC. After excluding 7 cases with incomplete medical records, we retrospectively 134 patients (115 men) who had undergone pulmonary metastasectomy for isolated PM of HCC. The enrollment of patients is shown in Fig. 1. The inclusion criteria of our study were as follows: (1) controlled state of primary HCC, (2) no other distant metastasis, and (3) appropriate pulmonary function test for major lung resection. All patients had available follow-up till December 2015.

### Methodology

Eighty-four patients underwent VATS (VATS group), and 50 underwent thoracotomy or sternotomy (open group). Under general anesthesia and double-lumen endotracheal tube insertion for single-lung ventilation, the patients’ chest that is where the lesion is located was up on the degree of 90°. Three or four surgical ports were created for VATS, and posterolateral thoracotomy or median sternotomy was performed for open surgery. Surgical approaches such as thoracotomy, sternotomy, and VATS were determined depending on the size, number, location, and laterality of the metastatic nodules. VATS can be applied for lesions of sizes below 3 cm, located in the outer one-third of the lung, having no endobronchial extension. The metastatic lesion located in the periphery of the lung was non-problematically resected by wedge resection. Segmentectomy or lobectomy was needed for deeply located lesions. Mediastinal lymph node dissections were performed only if enlarged lymph nodes were revealed on chest CT. This study was approved by the Asan Medical Center Ethics Committee/Review Board (2019-1166).

### Statistical analysis

Time-to-pulmonary recurrence after the initial treatment (TPR) was defined as the interval between control of HCC and the diagnosis of PM. Disease-free survival (DFS) after the first pulmonary metastasectomy was defined as the interval between the first pulmonary metastasectomy and diagnosis of a lesion related to HCC after the first pulmonary metastasectomy. Pulmonary DFS after metastasectomy (PDFS) was defined as the interval between the first pulmonary metastasectomy and the diagnosis of the second PM. Overall survival time was measured by comparing the date of the first pulmonary metastasectomy to the date of death or the last follow-up. Preoperative alpha-fetoprotein (AFP) was defined as the value of the latest serum AFP level measured before the first metastasectomy. Postoperative AFP was defined as the value of the earliest measured serum AFP level after the first metastasectomy, within 2 months. The calculation of the BCLC stage was based on before lung metastasis was detected. This is because, based on the time after lung metastasis is found, all patients are at BCLC stage c or higher, and other variables of the patient before surgery may not be considered. Categorical variables were expressed as frequencies and percentages and were compared between the two approaches using the chi-square test or Fisher’s exact test. Continuous variables were shown as means with standard deviations or median values with range and were compared using a Student’s *t* test. Univariate and multivariate analyses for prognostic factors were performed using Cox’s proportional hazard model. Survival rates were estimated using the Kaplan-Meier method, and survival according to the prognostic factors was compared using a log-rank test. Cox proportional hazard models were used to identify the predictors of mortality and recurrence. After excluding the correlated variables, independent variables with *p* values ≤ 0.05 from the univariate analysis were used for the initial multivariate Cox. The final multivariable model was selected using forward stepwise selection (*p* value ≤ 0.10 for entering the model and *p* ≤ 0.05 for staying in the model).

There were several considerations (baseline characteristics, HCC factors, surgical factors) for PSM; 13 variables (age, sex, history of liver cirrhosis, level of initial AFP, etiology of HCC, BCLC stage of HCC, Child-Pugh score, Eastern Cooperative Oncology Group (ECOG) scale of performance status, treatment method for primary HCC, local recurrence or progression of HCC, number of PM, maximum diameter of PM, TPR) were used for PSM. For PSM, observation pairs with equivalent propensity scores were selected with nearest-neighbor matching and a caliper width of 0.2 standard deviation. Patients in the VATS group were randomly matched to patients in the open group at a ratio of 1:1. The final sample consisted of 32 matched patients in each group.

Statistical analyses were performed using SPSS version 21.0 (SPSS Inc. Armonk, NY, USA) and R version 3.4.2 (R Project for Statistical Computing, Vienna, Austria). A value of *p* < 0.05 was considered statistically significant.

## Results

The median age was 55.0 years (range, 24–76 years). Patient characteristics are summarized in Table 1.

**Table 1:** Patients’ baseline and primary hepatocellular carcinoma characteristics

	Total (***n*** = 134)	Open (***n*** = 50)	VATS (***n*** = 84)	***p*** value
Age (years)	54.4 ± 9.5	53.1 ± 9.2	55.1 ± 9.7	0.261
Sex (male)	115 (85.8%)	45 (90.0%)	70 (83.3%)	0.285
BCLC stage				0.206
0	4 (3.0%)	0	4 (4.8%)	
A	34 (25.4%)	15 (30.0%)	19 (22.6%)	
B	49 (36.6%)	18 (36.0%)	31 (36.9%)	
C	43 (32.1%)	14 (28.0%)	29 (34.5%)	
D	4 (3.0%)	3 (6.0%)	1 (1.2%)	
ECOG performance status				0.571
0	112 (83.6%)	41 (82.0%)	71 (84.5%)	
1	17 (12.7%)	6 (12.0%)	11 (13.1%)	
2	4 (3.0%)	2 (4.0%)	2 (2.4%)	
3	1 (0.7%)	1 (2.0%)	0	
4	0	0	0	
Etiology				0.716
HBV	111 (82.8%)	42 (84.0%)	69 (82.1%)	
HCV	1 (0.7%)	0	1 (1.2%)	
Alcohol	5 (3.7%)	1 (2.0%)	4 (4.8%)	
Unknown	17 (12.7%)	7 (14.0%)	10 (11.9%)	
Child-Pugh classification				0.422
A	116 (86.6%)	41 (82.0%)	75 (89.3%)	
B	13 (9.7%)	6 (12.0%)	7 (8.3%)	
C	5 (3.7%)	3 (6.0%)	2 (2.4%)	
Liver cirrhosis	49 (36.6%)	14 (28.0%)	35 (41.7%)	0.112
Initial AFP (ng/ml)	73.0 (1.0–585,000)	60.7 (1.0–585,000)	80.0 (1.0–485,000)	0.601
Treatment of HCC				0.171
Surgery	107 (79.9%)	43 (86.0%)	64 (76.2%)	
TACE	27 (20.1%)	7 (14.0%)	20 (23.8%)	
RFA	0	0	0	

*VATS* video-assisted thoracic surgery, *AFP* alpha-fetoprotein, *HBV* hepatitis B virus, *HCV* hepatitis C virus, *BCLC* Barcelona Clinic Liver Cancer, *PSM* propensity score matching, *ECOG* Eastern Cooperative Oncology Group, *TACE* transarterial chemoembolization, *RFA* radiofrequency ablation

Surgical treatment for primary HCC was performed in 107 patients (79.9%), liver cirrhosis was reported in 49 patients (36.6%), and local recurrence of HCC was found in 89 patients (66.4%). There were no significant differences in baseline and primary HCC characteristics between the VATS and open groups. Unilateral PM was found in 104 patients (77.6%), and the median number of PMs was 1.0 (range, 1–11). There was a significant difference in the size and number of metastatic nodules between the VATS and open groups (*p* = 0.039 and 0.009, respectively). Wedge resection was more frequently performed in the VATS group than in the open group. There was no operative mortality. The median overall survival time was 38.7 months (range, 4.4–172.3), and median TPR was 19.1 months (range, 5–95.9). Perioperative complications occurred in 6 (4.5%). Three patients had prolonged pleural effusion needing insertion of the chest tube, 2 patients had prolonged air leakage, and 1 patient had postoperative pneumonia. The median hospital days after metastasectomy was 5 days (range, 3–26), 4.0 days (range, 3–24) in the VATS group, and 7.0 days (range, 4–26) in the open group (*p* = 0.001) (Table 2).

**Table 2:** Characteristics of the first pulmonary metastasectomy

	Total (***n*** = 134)	Open (***n*** = 50)	VATS (***n*** = 84)	***p*** value
Preoperative AFP (ng/ml)	18.9 (1.1–12,000.0)	34.9 (1.1–7330.0)	9.9 (1.2–12000.0)	0.155
Postoperative AFP (ng/ml)	10.6 (0–28,200.0)	11.8 (0–28,200.0)	8.8 (0–18,800.0)	0.193
Local recurrence	73 (50.3%)	33 (66.0%)	40 (47.6%)	0.112
Progression of local disease	16 (11.0%)	5 (10.0%)	11 (13.1%)	
Laterality				0.039
Bilateral	30 (22.4%)	16 (32.0%)	14 (16.7%)	
Unilateral	104 (77.6%)	34 (68.0%)	70 (83.3%)	
Number of metastasis	1.8 ± 1.4	2.2 ± 1.9	1.5 ± 0.9	0.009
Size (mm)	14.9 ± 9.4	16.4 ± 8.1	14.0 ± 10.0	0.143
Extent of resection				0.001
Wedge resection	104 (77.6%)	30 (60.0%)	74 (88.1%)	
Segmentectomy/lobectomy	30 (22.4%)	20 (40.0%)	10 (11.9%)	
Hospital stay (days)	6.4 ± 4.2	7.9 ± 4.0	5.4 ± 4.1	0.001
Complications	6 (4.5%)	4 (8.0%)	2 (2.4%)	0.195
DFI from HCC (months)	22.6 ± 17.2	21.8 ± 16.4	23.1 ± 17.7	0.666
DFI from PM (months)	22.3 ± 39.4	22.6 ± 43.7	22.2 ± 36.9	0.945
Overall survival time (months)	52.1 ± 41.0	56.4 ± 46.6	49.5 ± 37.3	0.377

*VATS* video-assisted thoracic surgery, *AFP* alpha-fetoprotein, *HCC* hepatocellular carcinoma, *PM* pulmonary metastasis, *DFI* disease-free interval

**Fig. 2:**
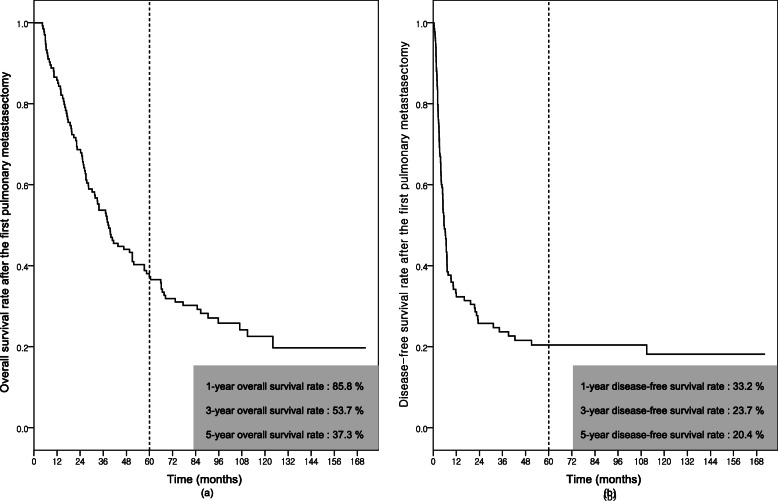
Survival rate from the first pulmonary metastasectomy in all patients; overall survival rate (**a**) and disease-free survival rate (**b**)

For the median 33.4 months (range, 1.8–112.0) duration of the follow-up from first metastasectomy, 113 (84.3%) patients experienced recurrence: 63 (55.7%) in the lung, 26 (23.0%) in the liver, 2 (1.8%) in the brain, 13 (11.5%) in other single organs, and 9 (8.0%) in multiple organs. Pulmonary-specific recurrences were observed in 95 (70.9%) patients. Forty-three (45.3%) patients had ipsilateral pulmonary metastases. Forty-five (47.4%) patients underwent repeated metastasectomies, 27 (28.4%) patients received chemotherapy, 4 (4.2%) patients received radiation therapy, 2 (2.1%) patients received chemoradiation therapy, and in 17 (17.9%) patients, metastatic lesions remained for various reasons (Table 3). One hundred patients (74.6%) died of disease progression. The 1-, 3-, and 5-year overall survival rates after the first pulmonary metastasectomy were 85.8%, 53.7%, and 37.3%, respectively. Disease-free survival rates after the first pulmonary metastasectomy were 33.2% in 1 year, 23.7% in 3 years, and 20.4% in 5 years, respectively (Fig. 2).

**Table 3:** Recurrence of metastasis from hepatocellular carcinoma after the first pulmonary metastasectomy

	Total (***n*** = 134)	Open (***n*** = 50)	VATS (***n*** = 84)	***p*** value
Recurrence after metastasectomy	113 (84.3%)	42 (84.0%)	71 (84.5%)	0.936
Recurred site				0.581
Lung	63 (47.0%)	27 (54.0%)	36 (42.9%)	
Liver	26 (19.4%)	9 (18.0%)	17 (20.2%)	
Bone	0	0	0	
Brain	2 (1.5%)	1 (2.0%)	1 (1.2%)	
Different single organ	13 (9.7%)	2 (4.0%)	11 (13.1%)	
Multiple organs	9 (6.7%)	3 (6.0%)	6 (7.1%)	
Pulmonary specific recurrence	95 (70.9%)	38 (76.0%)	57 (67.9%)	0.316
Recurred site				0.297
Ipsilateral	43 (32.1%)	20 (40.0%)	23 (27.4%)	
Contralateral	52 (38.8%)	18 (36.0%)	34 (40.5%)	
Treatment of recurred PM				0.533
Surgery	45 (33.6%)	18 (36.0%)	27 (32.1%)	
Chemotherapy	27 (20.1%)	10 (20.0%)	17 (20.2%)	
Radiotherapy	4 (3.0%)	2 (4.0%)	2 (2.4%)	
Chemoradiation	2 (1.5%)	2 (4.0%)	0	
Observation	17 (12.7%)	6 (12.0%)	11 (13.2%)	

*VATS* video-assisted thoracic surgery, *PM* pulmonary metastasis

Local recurrence of HCC, history of liver cirrhosis, and preoperative AFP were found to be independent prognostic factors for overall survival (Table 4). Local recurrence of HCC and TPR were independent prognostic factors for DFS after the first pulmonary metastasectomy (*p* < 0.001 and 0.005, respectively) (Table 5). For pulmonary-specific recurrence, local recurrence of HCC and preoperative AFP were independent prognostic factors (*p* < 0.001 and *p* = 0.013, respectively) (Table 6).

**Table 4:** Univariate and multivariate analysis for overall survival after the first pulmonary metastasectomy

Characteristics	Univariate	Multivariate	
	***p*** value	HR (95% CI)	***p*** value
Age > 50 years	0.375		
Liver cirrhosis (+)	0.051	1.729 (1.142–2.619)	0.010
Hepatitis viral marker (+)	0.457		
BCLC stage ≥ C	0.035		
ECOG performance status ≥1	0.089		
Child-Pugh score ≥ B	0.025		
Local recurrence of HCC	< 0.001	2.495 (1.571–3.963)	< 0.001
Initial AFP > 500 ng/ml	0.318		
Preoperative AFP > 500 ng/ml	< 0.001	2.632 (1.554–4.456)	< 0.001
Wedge resection as metastasectomy	0.423		
Multiple PM	0.274		
Maximum diameter of PM > 2 cm	0.130		
Hospital days ≥ 7	0.093		
Time-to-pulmonary recurrence period	0.113		
Open metastasectomy	0.870		

*HCC* hepatocellular carcinoma, *HR* hazard ratio, *CI* confidence interval, *AFP* alpha-fetoprotein, *BCLC* Barcelona Clinic Liver Cancer, *ECOG* Eastern Cooperative Oncology Group, *PM* pulmonary metastasis

**Table 5:** Univariate and multivariate analysis for disease-free survival after the first pulmonary metastasectomy

Characteristics	Univariate	Multivariate	
	***p*** value	HR (95% CI)	***p*** value
Age > 50 years	0.464		
Liver cirrhosis (+)	0.173		
Hepatitis viral marker (+)	0.627		
BCLC stage ≥ C	0.072		
ECOG performance status ≥1	0.161		
Child-Pugh score ≥ B	0.120		
Local recurrence of HCC	< 0.001	2.717 (1.694–4.357)	< 0.001
Initial AFP > 500 ng/ml	0.910		
Preoperative AFP > 500 ng/ml	0.037		
Wedge resection as a metastasectomy	0.199		
Multiple PM	0.265		
Maximum diameter of PM > 2 cm	0.387		
Hospital days ≥ 7	0.044		
Time-to-pulmonary recurrence period	0.068	1.852 (1.204–2.848)	0.005
Open metastasectomy	0.943		

*HCC* hepatocellular carcinoma, *HR* hazard ratio, *CI* confidence interval, *AFP* alpha-fetoprotein, *BCLC* Barcelona Clinic Liver Cancer, *ECOG* Eastern Cooperative Oncology Group, *PM* pulmonary metastasis

**Table 6:** Univariate and multivariate analysis for pulmonary-specific disease-free survival after the first pulmonary metastasectomy

Characteristics	Univariate	Multivariate	
	***p*** value	HR (95% CI)	***p*** value
Age > 50	0.253		
Liver cirrhosis (+)	0.216		
Hepatitis viral marker (+)	0.628		
BCLC stage ≥ C	0.023		
ECOG performance status ≥1	0.020		
Child-Pugh score ≥ B	0.156		
Local recurrence of HCC	< 0.001	3.105 (1.946–4.954)	< 0.001
Initial AFP > 500 ng/ml	0.955		
Preoperative AFP > 500 ng/ml	0.082	1.978 (1.157–3.382)	0.013
Wedge resection as metastasectomy	0.227		
Multiple PM	0.156		
Maximum diameter of PM > 2 cm	0.847		
Hospital days ≥7	0.086		
Time-to-pulmonary recurrence period	0.151		
Open metastasectomy	0.578		

*HCC* hepatocellular carcinoma, *HR* hazard ratio, *CI* confidence interval, *AFP* alpha-fetoprotein, *BCLC* Barcelona Clinic Liver Cancer, *ECOG* Eastern Cooperative Oncology Group, *PM* pulmonary metastasis

**Fig. 3:**
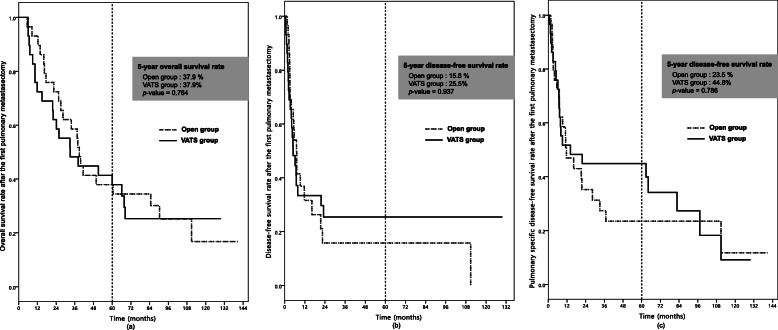
Survival rate after the first pulmonary metastasectomy in matched group; overall survival rate (**a**), disease-free survival rate (**b**), pulmonary specific disease-free survival rate (**c**)

The patients’ characteristics after PSM are summarized in Table 7. No significant differences in baseline and PM characteristics were observed. The open group underwent more extensive surgery and stayed more days than the VATS group (*p* = 0.025 and *p* = 0.017, respectively) (Table 8). There was no significant difference in overall survival rate, DFS rate, and PDFS rate after the first pulmonary metastasectomy between the VATS and open groups (*p* = 0.764, 0.937, and 0.786, respectively) (Fig. 3).

**Table 7:** Baseline characteristics of open and VATS group after propensity score matching

	Total (***n*** = 58)	Open (***n*** = 29)	VATS (***n*** = 29)	***p*** value
Age (years)	54.0 ± 11.1	54.8 ± 9.3	53.2 ± 12.7	0.606
Sex (male)	51 (87.9%)	26 (89.7%)	25 (86.2%)	0.687
BCLC stage				0.284
0	0	0	0	
A	13 (22.4%)	9 (31.0%)	4 (13.8%)	
B	21 (36.2%)	9 (31.0%)	12 (41.4%)	
C	24 (41.4%)	11 (38.0%)	13 (44.8%)	
D	0	0	0	
ECOG performance status				0.339
0	49 (84.5%)	24 (82.8%)	25 (86.2%)	
1	7 (12.1%)	3 (10.3%)	4 (13.8%)	
2	2 (3.4%)	2 (6.9%)	0	
3	0	0	0	
4	0	0	0	
Etiology				0.362
HBV	46 (79.3%)	24 (82.8%)	22 (75.9%)	
HCV	1 (1.7%)	0	1 (3.4%)	
Alcohol	2 (3.4%)	0	2 (6.9%)	
Unknown	9 (15.5%)	5 (17.2%)	4 (13.8%)	
Child-Pugh classification				0.706
A	50 (86.2%)	24 (82.8%)	26 (89.7%)	
B	8 (13.8%)	5 (17.2%)	3 (10.3%)	
C	0	0	0	
Liver cirrhosis	19 (32.8%)	11 (37.9%)	8 (27.6%)	0.401
Treatment of HCC				0.730
Surgery	48 (82.8%)	23 (89.3%)	25 (86.2%)	
TACE	10 (17.2%)	6 (20.7%)	4 (13.8%)	
RFA	0	0	0	

*VATS* video-assisted thoracic surgery, *AFP* alpha-fetoprotein, *HBV* hepatitis B virus, *HCV* hepatitis C virus, *BCLC* Barcelona Clinic Liver Cancer, *PSM* propensity score matching, *ECOG* Eastern Cooperative Oncology Group, *TACE* transarterial chemoembolization, *RFA* radiofrequency ablation

**Table 8:** Surgical characteristics of open and VATS group after propensity score matching

	Total (***n*** = 58)	Open (***n*** = 29)	VATS (***n*** = 29)	***p*** value
Preoperative AFP (ng/ml)	6.9 (0.6–12,000.0)	11.6 (1.1–6610.0)	4.7 (0.6–12,000.0)	0.981
Local recurrence	36 (62.1%)	17 (58.6%)	19 (65.5%)	0.373
Progression of local disease	5 (8.6%)	4 (13.8%)	1 (3.4%)	
Laterality				0.570
Unilateral	40 (69.0%)	19 (65.5%)	21 (72.4%)	
Bilateral	18 (31.0%)	10 (34.5%)	8 (27.6%)	
Number of metastasis	1.9 ± 1.4	2.1 ± 1.6	1.8 ± 1.3	0.414
Size (mm)	15.8 ± 8.8	16.9 ± 9.3	14.7 ± 8.2	0.343
Extent of resection				0.025
Wedge resection	46 (79.3%)	19 (65.5%)	27 (93.1%)	
Segmentectomy/lobectomy	12 (20.7%)	10 (34.5%)	2 (6.9%)	
Hospital stay (days)	6.2 ± 3.7	7.4 ± 3.0	5.1 ± 3.9	0.017
Complications	1 (1.7%)	1 (3.4%)	0	0.313
DFI from HCC (months)	24.0 ± 19.0	21.5 ± 16.0	26.5 ± 21.5	0.312
DFI from PM (months)	16.4 ± 28.0	14.9 ± 26.2	17.9 ± 30.1	0.683
Recurrence after metastasectomy	51 (87.9%)	27 (93.1%)	24 (82.8%)	0.227
Recurrence including PM	40 (69.0%)	23 (79.3%)	17 (58.6%)	0.089
Overall survival time (months)	49.2 ± 37.4	52.5 ± 38.1	46.0 ± 37.0	0.513

*VATS* video-assisted thoracic surgery, *AFP* alpha-fetoprotein, *HCC* hepatocellular carcinoma, *PM* pulmonary metastasis, *DFI* disease-free interval

## Discussion

The survival rate of HCC patients has markedly improved as a result of advances in surgical techniques and perioperative management. Although the lung is the most frequent site of extrahepatic metastasis from HCC, the role of surgery for PM from HCC has not been fully clarified yet, unlike PM from colorectal cancer or osteosarcoma. There have been some reports favoring surgical resection for isolated PM from HCC, and the present study supports those results [8–10]. While median survival time and 1-year survival rate of the patients with PM treated with chemotherapy were 4.6–14 months and 20–42%, respectively [5, 8], 5-year survival rate of patients who had undergone metastasectomy was reported to be 24–41.4% [15–20].

In the present study, the 5-year DFS rate and 5-year survival rate were 20.4% and 37.3%, respectively. More long-term survivors could be found among the patients who underwent metastasectomy than among those who received chemotherapy. However, since most studies were non-randomized and included only operable and resectable lesions in the surgery group, a comparison of outcomes between metastasectomy and chemotherapy needs circumspection.

Cheng et al. reported the overall survival of patients with advanced HCC with or without sorafenib. The median overall survival period was 6.5 and 4.2 months [7]. In our study, the median overall survival period was 38.7 months (range, 4.4–172.3). There are quite a few differences between the two studies. The reason being that the previous study included patients who were not subjected to surgery.

In this series, 81 (60.4%) patients had a single PM, while only 6 (4.5%) patients had five or more PMs. This implies that patients with a less invasive disease could be selected for metastasectomy. One hundred and thirty patients with four or less PMs showed a median survival period of 40.7 months (range, 4.4–172.3) regardless of the number of PMs. To evaluate the efficacy of metastasectomy, large randomized studies are needed.

Surgical intervention for PM can provide excellent local control. Due to its low mortality and morbidity [15–20], metastasectomy should be considered as a viable option to prevent pulmonary complications caused by PM. Several studies suggest that to improve long-term survival, patients with isolated PM and no other distant metastasis should be considered not only for surgical resection but also for repeated resection of PM [21–23]. In our experience, patients who underwent pulmonary metastasectomy could be discharged within approximately 7 days without operative mortality or serious complications. The second or third pulmonary metastasectomy could also be safely performed, and repeated metastasectomy could be attempted in patients with localized PM from HCC for better survival (5-year survival rate was 59.1% in repeated metastasectomy patients and 48.1% in non-surgical treatment patients, *p* = 0.002).

Introduction of VATS makes metastasectomy more endurable for patients with chronic diseases such as liver cirrhosis. Less pain and shorter hospitalization can lead to earlier returns to preoperative activities or subsequent treatment [11–13, 24]. However, the inability to thoroughly palpate the lung by VATS raises concerns about incomplete metastasectomy [25, 26] which could result in significantly worse survival rates [27]. In contrast, there are reports suggesting that the outcomes of thoracoscopic surgery are not inferior to those of open thoracotomy [13, 28]. This disagreement can partly be due to the possibility that finger palpation of the lung also has limitations in terms of detecting small nodules; in addition, metastasectomy under a thoracotomy is not always complete [29]. Recently, in contrast to the inaccuracy of old-generation CT, the 1-mm-thin section 16-channel multi-detector row CT showed a high detection rate of metastatic pulmonary nodules, especially in patients with non-osteosarcoma. Therefore, it can be a possible substitute for manual palpation [14]. If diagnosis by either imaging or palpation is incomplete, VATS should be considered for metastasectomy. VATS can minimize adhesion formation and render repeated resection more amenable. Given that complete resection is most important in any situation, conversion to thoracotomy should be promptly considered if the lesions detected by CT cannot be identified or resected by VATS. In the present study, all preoperatively detected PMs were completely resected either by VATS or by open surgery. Hospitalization duration and complication rate were significantly reduced in the VATS group. However, because more segmentectomies and lobectomies were adapted in the open group, it is difficult to compare the two methods straightforwardly. To overcome the shortcomings outlined above, in the present study, the propensity score matching analysis was adapted to compare two methods, and relatively favorable results were observed. However, matched analysis needs to drop unmatched data, for which, the main drawback is the shortage of cases. For this reason, further randomized large cohort studies need to be conducted in the future.

In the published literature, only several studies have reported risk factors related to long-term survival. Complete resection is the most significant predictor of better survival [18–20, 27]. Worse survival can be expected for patients with the factors causing a rise in the possibility of incomplete resection including multiple lesions [16, 30] and the factors predicting aggressive disease, such as short disease-free interval [18–20, 27, 30–33], multiple PMs, and higher serum AFP levels [19, 20]. It was suggested that the size of nodules had an inverse correlation with survival in PM from cancer other than HCC [22]. In the present study, local recurrence or progression of HCC, history of liver cirrhosis, and preoperative AFP were found to be independent prognostic factors for overall survival after the first pulmonary metastasectomy. Local recurrence or progression of HCC and preoperative AFP were also found to be independent prognostic factors for pulmonary-specific recurrence. Therefore, our results suggest that if there is a local recurrence or progression of HCC or if the levels of AFP before surgery are high, decisions on surgical treatments should be made with caution. The impact of all these proposed factors needs to be verified in future large-scale studies.

## Conclusion

When metastasis confined to the lung with the HCC under control or controllable, metatasectomy can be positively considered for patients with sufficient pulmonary reserve and in a good general condition. Local recurrence or progression of HCC, history of liver cirrhosis, and preoperative AFP were found to be independent prognostic factors. If there is a local recurrence or progression of HCC or if the level of AFP before metastasectomy is high, decisions on surgical treatment should be made with caution.

According to the results of the propensity score matching analysis, VATS metastasectomy provided outcomes comparable to those afforded by open metastasectomy. The most important prerequisite for pulmonary metastasectomy is complete resection.

## Data Availability

The datasets during and/or analyzed during the current study are available from the corresponding author on reasonable request.

## Abbreviations

HCC: Hepatocellular carcinoma
PM: Pulmonary metastasis
SHARP: Sorafenib HCC Assessment Randomized Protocol
HR: Hazard ratio
VATS: Video-assisted thoracic surgery
CT: Computed tomography
TPR: Time-to-pulmonary recurrence
DFS: Disease-free survival
PDFS: Pulmonary DFS after metastasectomy
AFP: Alpha-fetoprotein
HBV: Hepatitis B virus
BCLC: Barcelona Clinic Liver Cancer
PSM: Propensity score matching
ECOG: Eastern Cooperative Oncology Group
